# Dexmedetomidine attenuates myocardial ischemia-reperfusion injury in vitro by inhibiting NLRP3 Inflammasome activation

**DOI:** 10.1186/s12871-021-01334-5

**Published:** 2021-04-06

**Authors:** Yaru Huang, Xiaotong Sun, Zhaodong Juan, Rui Zhang, Ruoguo Wang, Shuqi Meng, Jiajia Zhou, Yan Li, Keyou Xu, Keliang Xie

**Affiliations:** 1 Shandong Provincial Medicine and Health Key Laboratory of Clinical Anesthesia, School of Anesthesiology, Weifang Medical University, No. 7166, Baotong West Street, Weicheng District, Weifang, 261021 China; 2 Department of Pain, Affiliated Hospital of Weifang Medical University, Weifang, 261000 China

**Keywords:** Cardiac fibroblasts, NLRP3 inflammasome, Dexmedetomidine, Hypoxia and reoxygenation injury

## Abstract

**Background:**

Myocardial ischemia-reperfusion injury (MIRI) is the most common cause of death worldwide. The NOD-, LRR- and pyrin domain-containing protein 3 (NLRP3) inflammasome plays an important role in the inflammatory response to MIRI. Dexmedetomidine (DEX), a specific agonist of α2-adrenergic receptor, is commonly used for sedation and analgesia in anesthesia and critically ill patients. Several studies have shown that dexmedetomidine has a strong anti-inflammatory effect in many diseases. Here, we investigated whether dexmedetomidine protects against MIRI by inhibiting the activation of the NLRP3 inflammasome in vitro.

**Methods:**

We established an MIRI model in cardiomyocytes (CMs) alone and in coculture with cardiac fibroblasts (CFs) by hypoxia/reoxygenation (H/R) in vitro. The cells were treated with dexmedetomidine with or without MCC950 (a potent selective NLRP3 inhibitor). The beating rate and cell viability of cardiomyocytes, NLRP3 localization, the expression of inflammatory cytokines and NLRP3 inflammasome-related proteins, and the expression of apoptosis-related proteins, including Bcl2 and BAX, were determined.

**Results:**

Dexmedetomidine treatment increased the beating rates and viability of cardiomyocytes cocultured with cardiac fibroblasts. The expression of the NLRP3 protein was significantly upregulated in cardiac fibroblasts but not in cardiomyocytes after H/R and was significantly attenuated by dexmedetomidine treatment. Expression of the inflammatory cytokines IL-1β, IL-18 and TNF-α was significantly increased in cardiac fibroblasts after H/R and was attenuated by dexmedetomidine treatment. NLRP3 inflammasome activation induced the increased expression of cleaved caspase1, mature IL-1β and IL-18, while dexmedetomidine suppressed H/R-induced NLRP3 inflammasome activation in cardiac fibroblasts. In addition, dexmedetomidine reduced the expression of Bcl2 and BAX in cocultured cardiomyocytes by suppressing H/R-induced NLRP3 inflammasome activation in cardiac fibroblasts.

**Conclusion:**

Dexmedetomidine treatment can suppress H/R-induced NLRP3 inflammasome activation in cardiac fibroblasts, thereby alleviating MIRI by inhibiting the inflammatory response.

**Supplementary Information:**

The online version contains supplementary material available at 10.1186/s12871-021-01334-5.

## Introduction

Acute myocardial infarction (AMI) represents a leading cause of death in patients with coronary heart disease [1]. Ischemic heart disease is the most common cardiovascular disease in the clinic. In patients with an increased risk of cardiovascular complications, perioperative AMI can occur in up to 5.0% of major noncardiac inpatient operations [2]. AMI is myocardial necrosis caused by acute and persistent ischemia and hypoxia of the coronary artery. Reperfusion therapy is the most important treatment for AMI. However, the final infarction area is affected not only by the duration of ischemia but also by further injury during reperfusion, which is called ‘reperfusion injury’. Experimental studies in AMI animal models suggest that lethal reperfusion injury accounts for up to 50% of the final size of the myocardial infarct [3]. Therefore, future efforts should focus on developing a new cardioprotective strategy for myocardial ischemia-reperfusion injury (MIRI). The pathogenesis of MIRI mainly involves the inflammatory response, apoptosis, calcium overload, and oxygen free radical production [4], among which inflammation plays an important role in the pathophysiology of MIRI [5]. One prominent and early mediator of inflammation in the process of MIRI is interleukin-1β (IL-1β). Caspase-1 is an IL-1β-converting enzyme (ICE) involved in the processing of pro-IL-1β into mature IL-1β and is activated within the inflammasome. Several studies have indicated that the NOD-like receptor protein 3 (NLRP3) inflammasome is involved in MIRI [6]. NLRP3 inflammasomes include NLRP3, apoptosis-related dot-like protein containing a CARD (ASC) and Caspase-1. The NLRP3 receptor protein contains three domains: PYD, NACHT, and LRR. The protein of ASC acts as a bridge connecting the receptor protein and the effector protein. NLRP3 activates Caspase-1 by interacting with ASC. Activated caspase-1 cleaves IL-1β into active IL-1β and promotes its release through an unknown mechanism. Similarly, the release of IL-18, another member of the IL-1 cytokine family, also depends on caspase-1. The formation and activation of NLRP3 inflammasome promotes further myocardial injury after cardiac ischemia reperfusion [7].. Cardiac fibroblasts (CFs) are the most abundant cell type in the adult human heart. Increasing evidence shows that the NLRP3 inflammasome is upregulated in CFs in MIRI and may be a significant contributor to infarct size expansion during MIRI [8, 9].

Dexmedetomidine (DEX), a highly selective α2-adrenergic receptor agonist, is widely used for sedation and analgesia in intensive care units (ICUs) or as an anesthetic adjuvant [10]. Several studies have demonstrated that DEX has a strong anti-inflammatory property [11] and could alleviate MIRI [12]. Some studies and clinical trials have revealed that inflammatory cytokines increase during MIRI [13–15]. However, the mechanisms by which DEX alleviates MIRI remain to be determined. This study aimed to further investigate whether DEX treatment can suppress NLRP3 inflammasome activation in CFs, thereby alleviating MIRI in vitro.

## Materials and methods

### Isolation and culture of cardiomyocytes (CMs) and CFs

The present study was approved by the Ethical Board of Weifang Medical University (Weifang, China). Primary cultures of neonatal Sprague-Dawley rat CMs and CFs were prepared from 1 to 3-day-old rat hearts. Ventricles of neonatal rats were separated, minced and washed with cold solution A [10 mM HEPES (free acid), 0.4% fetal equine serum (HS), D-Hanks]. The heart tissue was dissociated using digestion solution B [200 U/ml collagenase II, 1% P/S (100 U/ml penicillin and 0.1 g/L streptomycin), solution A] in an Erlenmeyer flask filled with glass beads. The flask was then placed in an oscillator at 37 °C. Cell suspensions were collected from two dissociations, and the mixture was centrifuged at 700 rpm for 5 min. The cells were then resuspended in Dulbecco’s modified Eagle’s medium (DMEM)/high glucose supplemented with 5% newborn bovine serum (NBS), 8% HS and 1% penicillin-streptomycin liquid (P/S). Next, we used a cell strainer to filter the cell suspensions and plated the cells onto 25 cm^2^ cell culture flasks. The cells were incubated for 60 min so that CFs attached preferentially to the bottom of the dishes. The nonadherent cells (mainly CMs) were collected, and the adherent cells (mainly CFs) were supplemented with DMEM/high glucose containing 10% fetal bovine serum (FBS) and 1% P/S. The collected CMs were immediately plated onto cell culture flasks at a density of 1 × 10^5^ cells/cm^2^ and then cultured with DMEM/high glucose containing 5% NBS, 8% HS, 1% P/S and 0.1 mM 5-bromo-2-deoxyuridine (BrdU). CFs grew to confluence and were then passaged. The cells were then plated at 37 °C in humid air with 5% CO_2_. The culture media of CMs and CFs were changed every 2 days. All cells in each group were collected for WB, qPCR, CCK-8 experiments. All experiments mentioned below were repeated five times performed on five different cardiac cell isolations.

### Coculture conditions and measurement of cell beating rate

**Fig. 1:**
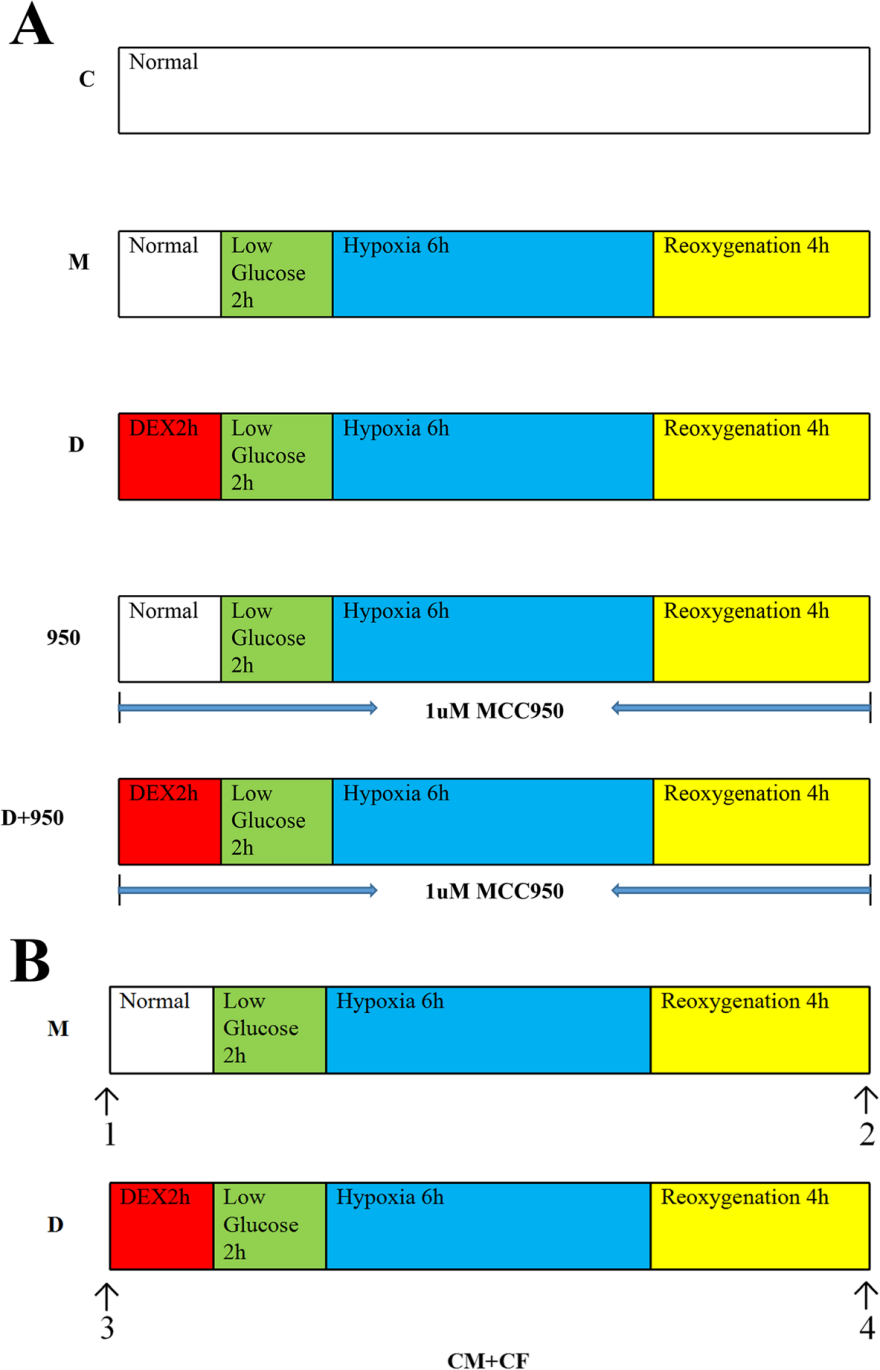
Experimental protocol and four time points for detecting the beating rate of cardiomyocytes (CMs). **a** Experimental protocols. Cardiac fibroblasts (CFs), cardiomyocytes (CMs) and cocultured CMs and CFs (CMs + CFs) were randomly assigned to one of five groups: 1) C group, in which the cells were incubated under normal conditions in a CO_2_ incubator; 2) M group, in which the cells were exposed to hypoxia/reoxygenation as we described above; 3) D group, in which the cells were pretreated with 1 μg/ml dexmedetomidine (DEX) two hours before hypoxia/reoxygenation; 4) 950 group, in which the cells were treated with 1 μM MCC950 during hypoxia/reoxygenation and two hours before it; and 5) D + 950 group, in which the cells were treated with 1 μM MCC950 during DEX preconditioning and hypoxia/reoxygenation,. **b** Four time points for detecting the myocardial cell beat frequency of cocultured CMs and CFs (CMs + CFs). We detected the beat frequency of CMs at these four time points as indicated by the arrow. 1–2 (group M): 1, normal; 2, after hypoxia/reoxygenation. 3–4 (group D): 3, normal; 4, after hypoxia/reoxygenation. DEX, dexmedetomidine; CMs, cardiomyocytes; CFs, cardiac fibroblasts; CMs + CFs, cocultured cardiomyocytes and cardiac fibroblasts; MCC950, a potent selective NLRP3 inhibitor

In the coculture experiments, CFs and CMs were mixed and plated at a ratio of 1:3 (25% CFs) on glass coverslips in 6-well or 96-well plates and glass bottom dishes [16]. One day after the cells attached to the bottom of the culture dishes, the beating rate of CMs at different time points was determined in each group by using a microscope (Fig. 1b). Beating rate of CMs were counted by one blinded from five fields of view under the microscope and presented as average.

### Hypoxia/reoxygenation (H/R) injury model

The model of H/R injury was established on day 4 after cell isolation. To simulate H/R injury, the cells were first cultured in serum-free DMEM/low (1000 mg/L glucose) glucose for 2 h under normoxic conditions (5% CO_2_, 95% air) in a CO_2_ incubator and then placed in a Tri-Gas (1% O_2_/5% CO_2_/94% N_2_) incubator. After 6 h of H/R exposure, the medium was replaced with medium with a normal glucose concentration (4500 mg/L glucose). The cells were incubated for an additional 4 h under normoxic conditions in a CO_2_ incubator before the assays below were performed.

### Experimental protocol

CFs, CMs and cocultures of CMs and CFs (CMs + CFs) were randomly assigned to one of five groups (Fig. 1a): 1) blank control group (group C), in which the cells were incubated under normoxic conditions in a CO_2_ incubator; 2) H/R group (group M), in which the cells were subjected to H/R as described above; 3) H/R + DEX group (group D), in which the cells were pretreated with 1 μg/ml DEX at 2 hours before H/R; 4) H/R + MCC950 group (group 950), in which the cells were treated with 1 μM MCC950 (Selleck, USA; a potent selective NLRP3 inhibitor) in the H/R period and the previous 2 hours; and 5) H/R + DEX + MCC950 group (group D + 950), in which the cells were treated with 1 μM MCC950 during DEX and H/R pretreatment.

### Cell viability analysis

Cell viability was determined using a Cell Counting Kit-8 (CCK-8) (Biosharp, Beijing) solution. Cells were seeded at a density of 1 × 10^4^ cells/well in 96-well plates and incubated for 24 to 48 h to observe the cell status. After the cells were subjected to H/R as described above, 10 μl CCK-8 (10 mg/ml) solution was added and incubated for 1 h. Absorbance was detected at 450 nm using a microplate reader.

### Western blot assay

Lysates were obtained by lysing the cell monolayer with phenylmethanesulfonyl fluoride (PMSF) and radioimmunoprecipitation assay (RIPA) lysis buffer (1:100). Total protein was separated in 12% sodium dodecyl sulfate polyacrylamide gel electrophoresis (SDS-PAGE) gels and then transferred to polyvinylidene difluoride (PVDF) membranes. Proteins were detected after labeling with specific primary antibodies [rabbit anti-NLRP3 (1:500, Bioss, China), rabbit anti-ASC polyclonal antibody (1:500, Bioss, China), rabbit anti-Caspase-1 (1:1000, Abcam, USA), rabbit anti cleaved Caspase1 (Arg317)/P10 (1:1000, Affinity, USA), rabbit anti-IL-1β antibody (1:1000, Abcam, USA), rabbit anti-BAX antibody (1:10000, Proteintech, USA), and rabbit anti-Bcl2 antibody (1:1500, Proteintech, USA)] followed by HRP-coupled secondary antibodies. The signals were detected by the addition of the chemiluminescent substrate (ECL Western Blot Kit, CWBIO, China). The protein amount was determined using Image J software. The level of GAPDH (1:1500, Proteintech, USA) was used to normalize the signal intensities.

### Supernatant IL-18 and TNF-α concentration measurement by ELISA

The concentration of TNF-α in the cell culture supernatant was measured by a rat TNF-α precoated ELISA kit (DAKEWE, China). Cell culture supernatant concentrations of IL-18 in rats were measured by a rat IL-18 ELISA kit (Boster, USA).

### Real-time PCR

Total RNA was isolated from CFs using TRIzol (Thermo Fisher Scientific, China), and real-time RT-PCR was conducted by a qPCR machine to determine mRNA expression. We followed the recommended steps for RNA extraction to extract total RNA from cells and measured the RNA concentration. We used a PCR machine to reverse transcribe RNA into cDNA according to the reagent instructions. For the qPCR experiments, we prepared 10 μl PCR master mix as follows: 4.8 μl cDNA template (15 ng/μl), 5 μl 2 × ChamQ SYBR qPCR Master Mix, and 0.2 μl gene-specific primers (10 μM). Then, we used a LightCycler 480 II (Roche, Indianapolis, IN, USA) to perform qPCR under the following conditions: predenaturation (5 min at 95 °C), followed by 40 cycles at 95 °C for 10 s and 60 °C for 30 s. The primer sets for the target genes are shown in Table 1.

**Table 1:** List of real-time RT-PCR primers

mRNA	Forward	Reserve
GAPDH	GTTACCAGGGCTGCCTTCTC	ACCAGCTTCCCATTCTCAGC
NLRP3	GGTGACCTTGTGTGTGCTTG	ATGTCCTGAGCCATGGAAGC
Caspase1	GACCGAGTGGTTCCCTCAAG	GACGTGTACGAGTGGGTGTT
ASC	GGACAGTACCAGGCAGTTCG	GTCACCAAGTAGGGCTGTGT
IL-1β	GGGATGATGACGACCTGC	CCACTTGTTGGCTTATGTT
IL-18	CAACCGCAGTAATACGGAGC	TCTGGTCTGGGATTCGTTGG
TNF-α	TGATCGGTCCCAACAAGGA	TGCTTGGTGGTTTGCTACGA

### Immunofluorescence

CFs cultured alone or in the presence of CMs were cultured on glass coverslips in 6-well plates. The cells were subjected to H/R as described above. Then, the cells were incubated with 4% fixative solution (Solarbio, China) for 30 min and blocked in 10% normal goat serum (Solarbio, China)/PBS for 30 min. The cells were incubated overnight at 4 °C with anti-NLRP3 (1:200), anti-ASC (1:200), anti-cleaved-caspase1 (1:200), anti-sarcomeric alpha actinin (1:200, Abcam, USA) and anti-Vimentin (1:200, Abcam, USA) primary antibodies. Thereafter, the cells were washed with PBS and incubated with goat anti-rabbit IgG/FITC (1:500, MultiSciences, China), goat anti-chicken IgG/Cy5 (1:500, Solarbio, China), and Cy3-conjugated Affinipure goat anti-mouse IgG (1:50, Proteintech, USA) secondary antibodies (30 min, room temperature) to visualize NLRP3, apoptosis-associated speck-like protein containing a CARD (ASC), cleaved caspase1, Vimentin and sarcomeric alpha actinin. We used 4′,6-diamidino-2-phenylindole (DAPI) (1:200, Beyotime, China) to visualize the nuclei. Finally, the fluorescence was visualized using a fluorescence microscope. Three fields of view were randomly selected to observe the fluorescent protein in each batch of cells by one blinded for allocation.

### Statistical analysis

SPSS statistical software (SPSS 20.0) was used by one blinded for allocation to perform statistical analyses. The data are represented as the mean ± standard deviation (SD). Statistically significant values were compared using One-way ANOVA, pairwise comparisons were determined by t test, and *P* < 0.05 was considered to indicate statistical significance.

## Results

### CFs treated with DEX increase the beating rates of cocultured CMs

**Fig. 2:**
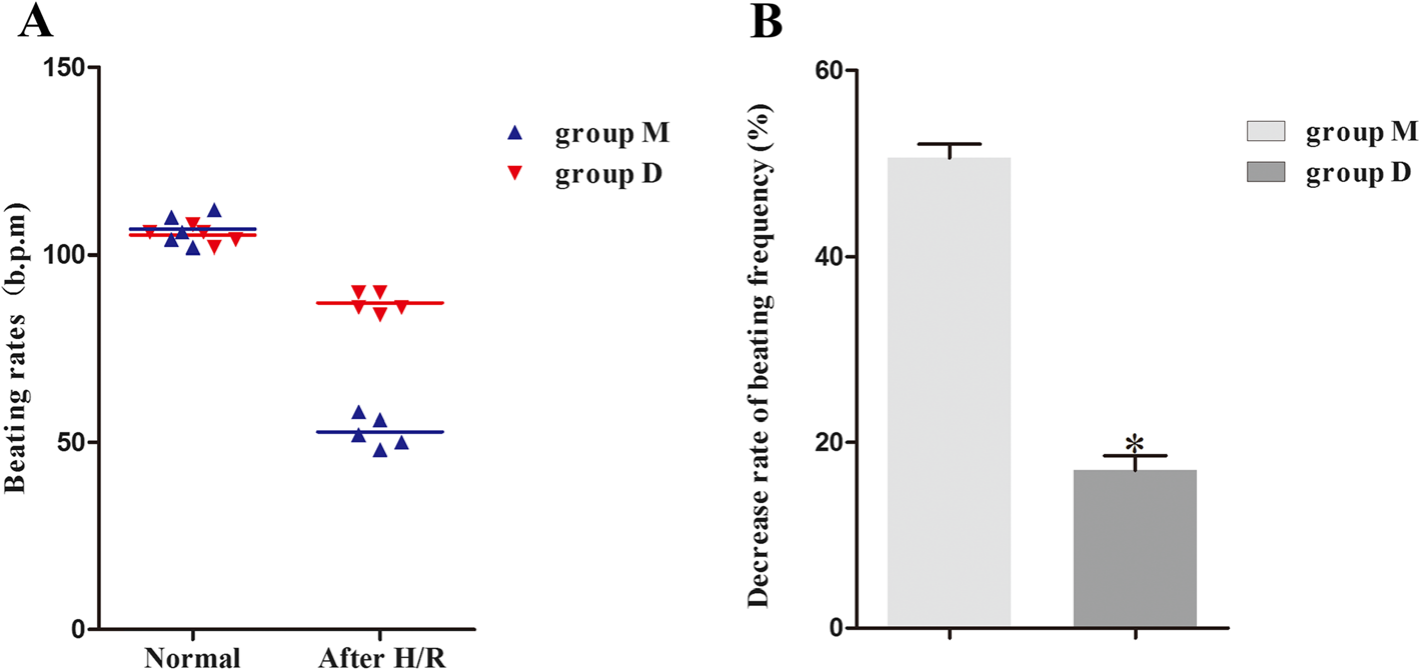
Comparison of the effect of DEX pretreatment on CM beating rates. **a** The beating rate of CMs in group M and group D of the cocultured CMs and CFs at normal times and after hypoxia/reoxygenation (H/R). **b** After H/R, the rate of decline in the beating rate of CMs pretreated with DEX was significantly lower than that of the other groups of cells. Decrease rate of beating frequency = (beating rate at the normal time - beating rate after H/R)/ beating rate at the normal time. Group M: cocultured CMs and CFs were subjected to H/R as described above. Group D: cocultured CMs and CFs were treated with 1 μg/ml DEX two hours before H/R. bpm, beats per minute. ^*^P < 0.05 vs. the M group; data are expressed as the means ± SD (*n* = 5) and analyzed by One-way ANOVA analysis of variance

As shown in Fig. 1b, we detected the beat frequency of CMs at the indicated four time points in cocultured CMs and CFs. The results are shown in Fig. 2. DEX pretreatment improved the beating rate of CMs in cocultures of CMs and CFs. After H/R, the rate of decline in the beating rate of group D was significantly lower than that of group M (group D 87 ± 3 b.p.m. vs. group M 52 ± 4 b.p.m., *p* < 0.05).

### DEX improves the cell viability affected by H/R

**Fig. 3:**
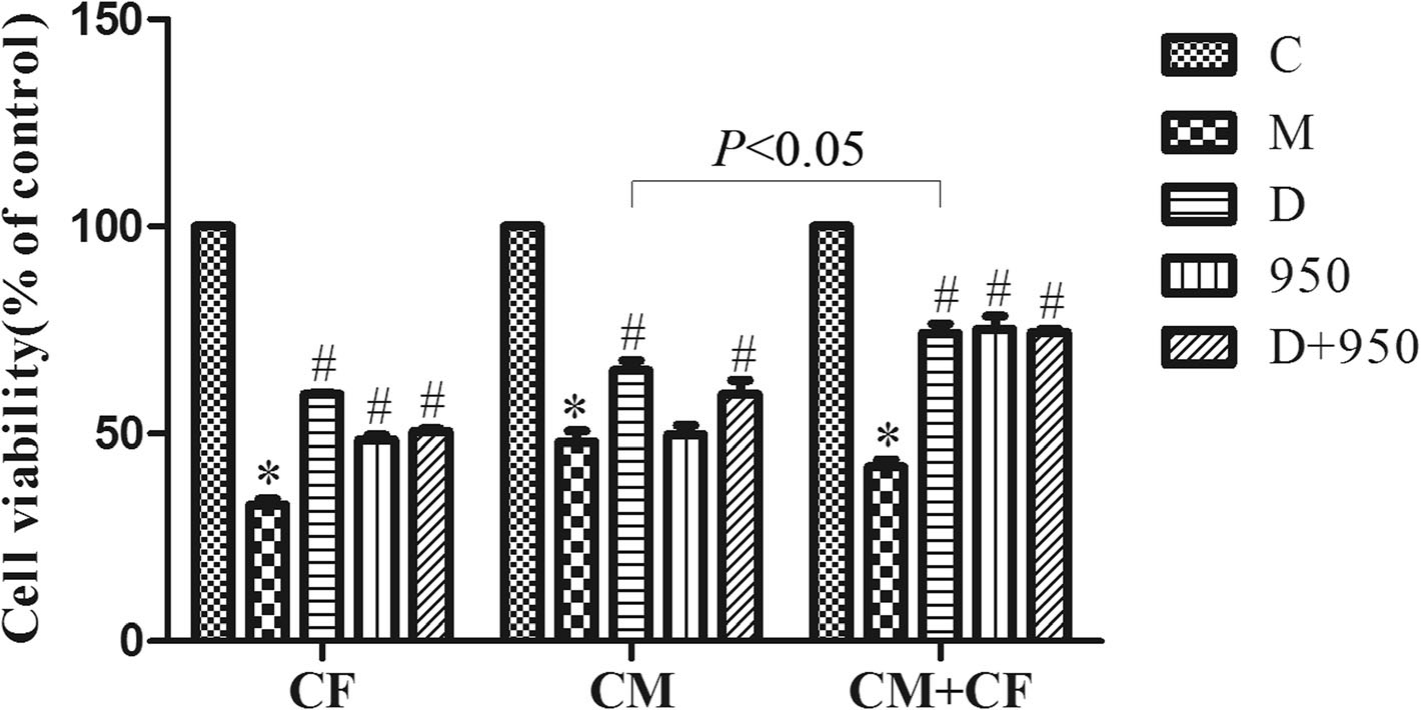
Effects of DEX on cell viability reduced by H/R injury. Cell viability was detected by the CCK-8 assay. ^*^P < 0.05 vs. the C group; ^*#*^*P* < 0.05 vs. the M group; data are expressed as the means ± SD (*n* = 5) and analyzed by One-way ANOVA analysis of variance. CMs, cardiomyocytes; CFs, cardiac fibroblasts; CMs + CFs, cocultured CMs and CFs

A CCK-8 assay was performed to evaluate the effect of DEX on H/R-induced decrease in cell viability in CMs, CFs and cocultured CMs and CFs. As evidenced by Fig. 3, the cell viability of the M groups was significantly lower than that of the corresponding C groups. Nevertheless, the CMs, CFs and cocultured CMs and CFs exposed to DEX had significantly increased cell viability compared with M group cells. For the D group, the viability of CMs in cocultured cells was significantly higher than that of CMs cultured alone. When the cells were treated with MCC950, an inhibitor of the NLRP3 inflammasome, the viability of CFs and cocultured cells was increased compared with that of M group cells but not CMs. When the cells were cotreated with MCC950 and DEX, the protective effect of DEX was similar to that of DEX pretreatment alone. Therefore, DEX improved cell viability by suppressing H/R-induced NLRP3 inflammasome activation in CFs.

### The NLRP3 protein is expressed in CFs rather than CMs

**Fig. 4:**
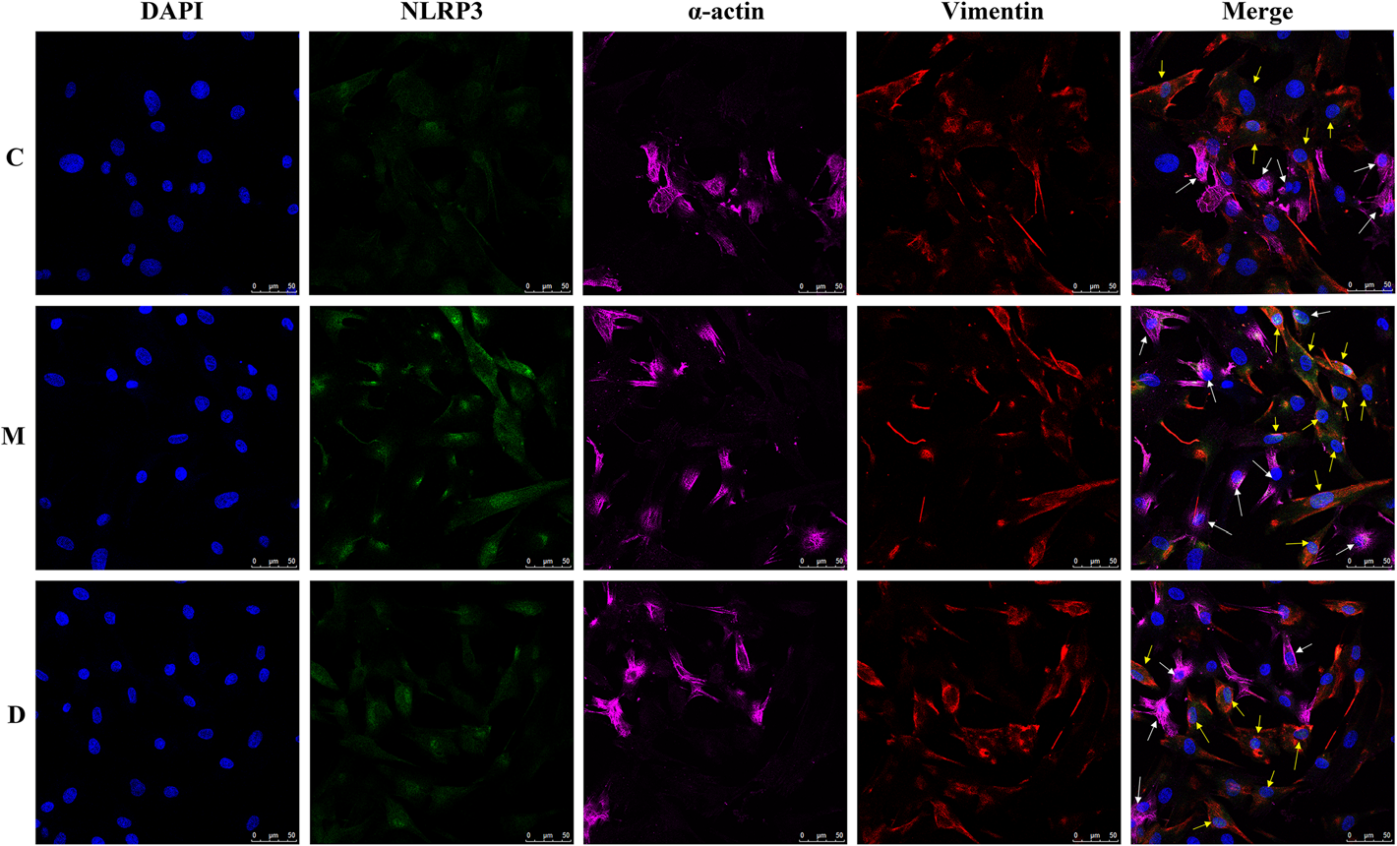
Localization and expression of NLRP3 in cocultured CFs and CMs. The protein expression of NLRP3, sarcomeric alpha actin and vimentin was observed in the C, M, and D groups (magnification, × 400) using a laser scanning confocal microscope. Sarcomeric alpha actin is a marker of CMs. Vimentin is a marker of CFs. Blue fluorescence indicates nuclear staining, green fluorescence indicates NLRP3 staining, purple fluorescence indicates sarcomeric alpha actinin staining (white arrow), and red fluorescence indicates vimentin staining (yellow arrow). CMs, cardiomyocytes; CFs, Cardiac fibroblasts

To verify the localization of NLRP3, the expression of NLRP3 in cocultures of CFs and CMs was determined by laser scanning confocal microscopy. As shown in Fig. 4, NLRP3 staining (green) coincided with vimentin staining (red). Therefore, the NLRP3 protein is expressed in CFs but not in CMs. At the same time, we also observed that the positive immune reaction of NLRP3 in the M group was enhanced compared with that in the C group. In addition, the immunoreactivity of NLRP3 was decreased in the D group compared to the M group.

### DEX relieves the inflammatory response induced by H/R

**Fig. 5:**
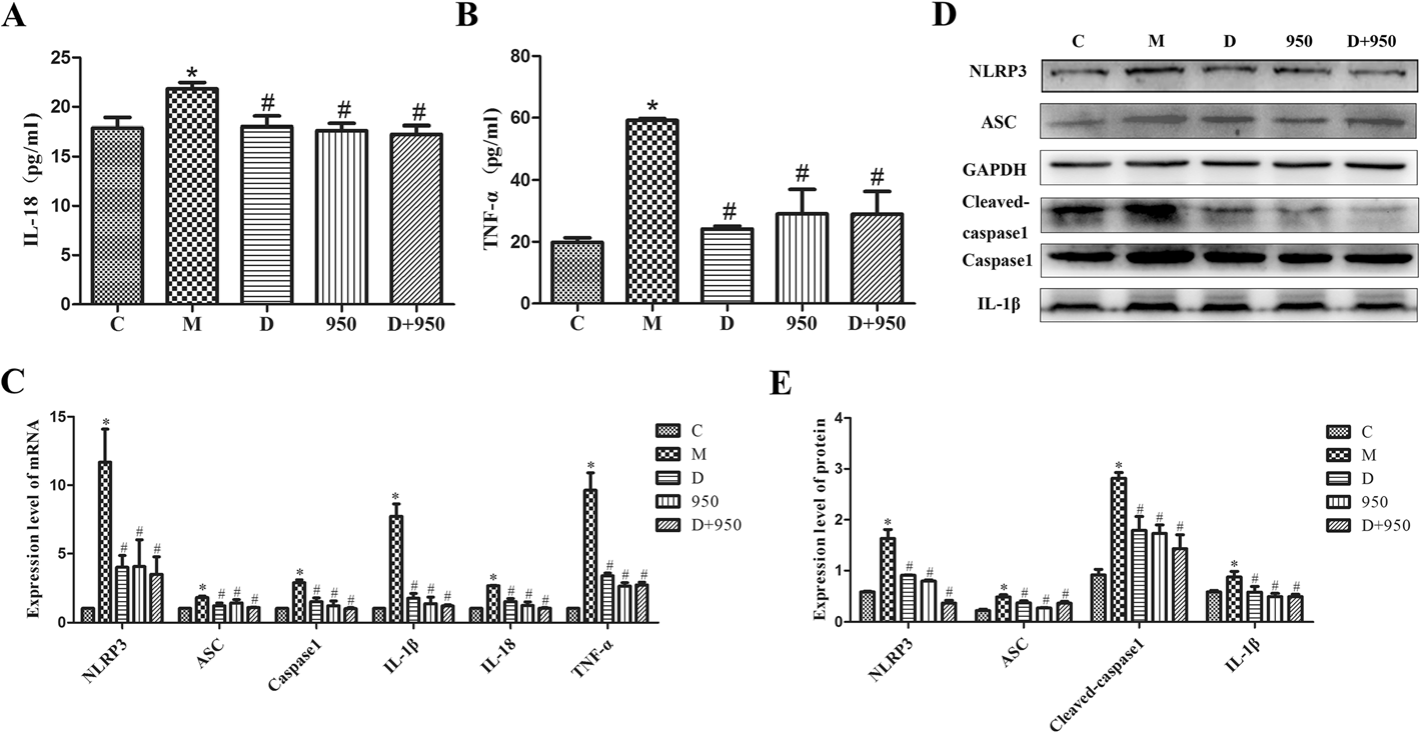
Effects of DEX on inflammation and the NLRP3 inflammasome in H/R injury. **a** The levels of TNF-α were determined in CF supernatant by ELISA. **b** The levels of IL-18 were determined in CF supernatant by ELISA. **c** The mRNA levels of IL-1β, IL-18, TNF-α, NLRP3, Caspase1, and ASC in CFs treated with DEX, MCC950 and DEX + MCC950 examined by RT-qPCR. **d** The densitometric value of NLRP3, Caspase1, cleaved caspase-1, ASC and IL-1β protein bands in CFs treated with DEX, MCC950 and DEX + MCC950 examined by western blot assay. Full-length blots are presented in Supplementary Fig. 1–6). **e** The protein levels of NLRP3, Caspase1, cleaved caspase-1, ASC and IL-1β in CFs treated with DEX, MCC950 and DEX + MCC950 examined by western blot analysis. ^*^P < 0.05 vs. the C group; ^*#*^*P* < 0.05 vs. the M group; data are expressed as the means ± SD (*n* = 5) and analyzed by One-way ANOVA analysis of variance

The levels of inflammatory cytokines were determined in CF supernatant and lysates. The results showed that H/R increased the levels of TNF-α and IL-18 in the CF supernatant (Fig. 5a, b) and the levels of IL-1β in CF lysates (Fig. 5e,), which were dramatically decreased in the D, 950, and D + 950 groups.

### DEX suppresses H/R-induced NLRP3 inflammasome activation in CFs

Next, we investigated the role of DEX in the activation of the NLRP3 inflammasome in CFs. We found that DEX, MCC950, and DEX + MCC950 suppressed H/R-induced NLRP3 inflammasome activation, as evidenced by a decrease in NLRP3, Caspase-1 and ASC mRNA levels (Fig. 5c).

### DEX reduces the levels of NLRP3 inflammasome-related proteins in CFs

To evaluate the effect of DEX on H/R-induced NLRP3 inflammasome activation, the levels of NLRP3 inflammasome-related proteins in CFs were determined by western blotting. As shown in Fig. 5d, e, the activity of NLRP3 was markedly increased in the M group. The inflammasome induces the maturation and release of IL-1β and IL-18 by activating Caspase-1, causing an inflammatory response, so the activity of caspase-1 in CFs was determined. Caspase-1 p10 (an autoprocessed fragment of Caspase-1) was markedly increased in the M group. In addition, the protein levels of ASC and IL-1β in CFs were significantly increased by H/R. However, DEX, MCC950, and DEX + MCC950 treatment decreased the levels of these proteins.

### Effects of DEX, MCC950, and DEX + MCC950 treatment on NLRP3, cleaved caspase1, and ASC protein levels in H/R CFs

**Fig. 6:**
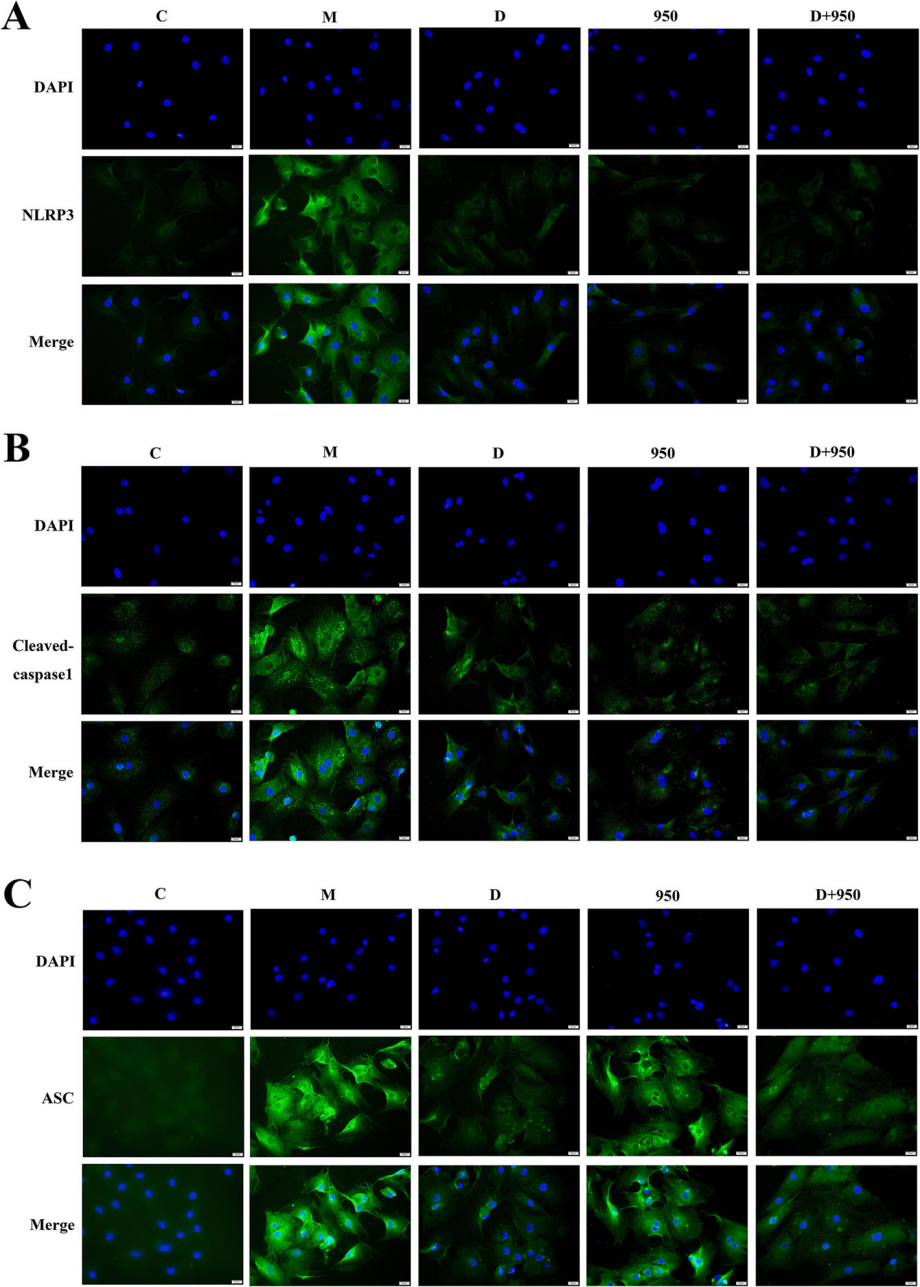
Localization and expression of NLRP3, cleaved caspase-1 and ASC in CFs. The protein expression of NLRP3, cleaved caspase1 and ASC was observed in the C, M, D, 950, and D + 950 groups (magnification, × 400). Blue fluorescence indicates nuclear staining, and green fluorescence indicates NLRP3, cleaved caspase1 or ASC staining

To further verify the effect of DEX on NLRP3 inflammasome activation, the expression of NLRP3 inflammasome-related proteins in CFs was determined by immunofluorescence. As shown in Fig. 6, compared with that in the C group, the positive immune reaction of NLRP3, cleaved caspase1 and ASC was enhanced in the M group. Furthermore, compared with that in the M group, the immunoreactivity of these proteins in the D, 950 and D + 950 groups was decreased. These results are consistent with the western blot data.

### DEX reduces cell apoptosis by suppressing H/R-induced NLRP3 inflammasome activation in CFs

**Fig. 7:**
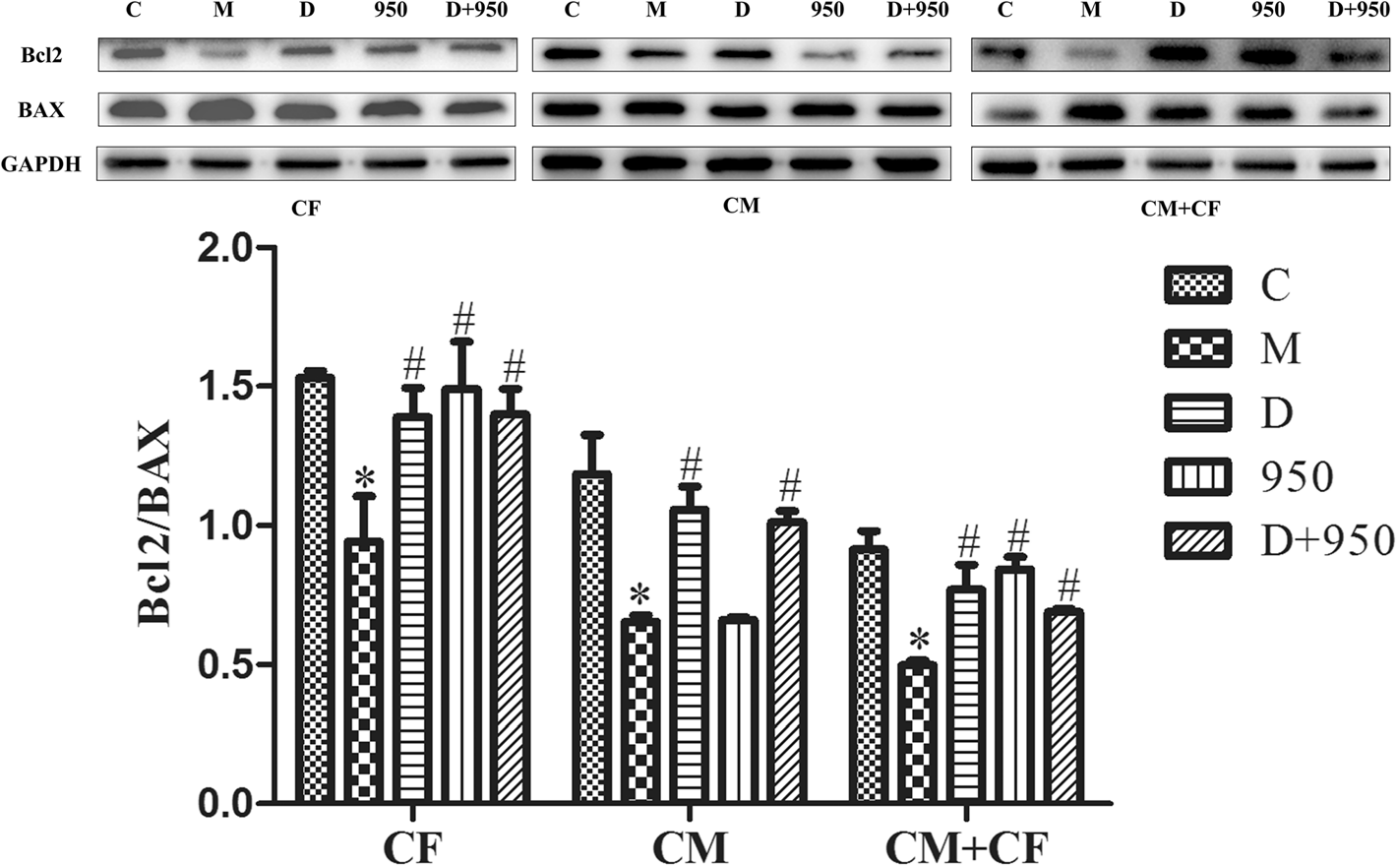
Effects of DEX on cell apoptosis induced by H/R injury. The protein levels of Bcl2 and Bax in CFs, CMs, and CMs + CFs were determined by western blot assay. Full-length blots are presented in Supplementary Fig. 7–15. ^*^P < 0.05 vs. the C group; ^*#*^*P* < 0.05 vs. the M group; data are expressed as the means ± SD (n = 5) and analyzed by One-way ANOVA analysis of variance. CM, cardiomyocytes; CF, cardiac fibroblasts; CMs + CFs, cocultured CMs and CFs

H/R increased the apoptosis of CMs, CFs and cocultured cells, while DEX decreased the apoptosis of the cells. The expression of Bcl2 and Bax was determined by western blot analysis. As shown in Fig. 7, Bax expression was increased and Bcl2 expression was decreased when the cells were exposed to H/R, while DEX pretreatment somewhat suppressed the expression of Bax and promoted the expression of Bcl2 in H/R-induced cells. When the cells were treated with MCC950, an inhibitor of the NLRP3 inflammasome, the apoptosis of CFs and cocultured cells, but not CMs, was reduced. When the cells were cotreated with MCC950 and DEX, the protective effect of DEX was similar to that of DEX pretreatment alone. Therefore, DEX suppressed cell apoptosis by suppressing H/R-induced NLRP3 inflammasome activation in CFs.

## Discussion

The main results of this study were as follows: (1) CFs pretreated with DEX improved the beat rates of cocultured CMs and cell viability reduced by H/R; (2) DEX improves the cell viability affected by H/R; (3) The NLRP3 protein was expressed in CFs rather than CMs; (4) The expression of IL-1β, IL-18, TNF-α, NLRP3, cleaved caspase1, and ASC was significantly upregulated in the CFs of group M; (5) DEX pretreatment decreased the expression of IL-1β, IL-18, TNF-α, NLRP3, cleaved caspase1, and ASC in the H/R model of CFs; and (6) DEX reduced the expression of BAX and promoted BAX by suppressing H/R-induced NLRP3 inflammasome activation in CFs. These findings suggest that DEX pretreatment can inhibit the activation of the NLRP3 inflammasome in CFs, reduce the inflammatory response of CFs and alleviate MIRI.

Several studies have shown that MIRI is associated with the NLRP3 inflammasome, and DEX can attenuate the inflammatory response to MIRI [8, 17]. In the present study, the H/R model was established with or without pretreatment with DEX. In corroboration with the results of previous studies, the present study demonstrated that H/R significantly reduced the viability of CFs, CMs and CMs + CFs and promoted apoptosis, whereas DEX pretreatment prior to H/R reduced H/R-induced damage in primary neonatal rat CMs [18]. This study also found that, compared with CMs alone, cocultured CMs and CFs pretreated with DEX showed improved cell viability, which was reduced after H/R (Fig. 3). We proceeded with CFs to further explore the effects of DEX on MIRI and its potential molecular mechanisms. This study confirmed that NLRP3 inflammasome activation in CFs plays an important role in H/R injury and that DEX can inhibit the NLRP3/Caspase-1/ASC signaling pathway to alleviate H/R injury. This provides more evidence for the use of DEX as a protective drug for MIRI.

CFs account for approximately 2/3 of the cardiac cellular population and play an important role in cardiac development and function [19]. Recent studies indicate that CFs can activate the inflammatory response following MIRI [20]. Increasing evidence demonstrates that CFs are the main resident cells that release IL-1β, and the NLRP3 inflammasome of CFs is an important mediator of MIRI [5]. By exploring the localization of the NLRP3 inflammasome in CMs and CFs, this study suggests that NLRP3 inflammasomes are mainly expressed in CFs rather than CMs (Fig. 4). The inflammatory response is increased during MIRI [13–15]. A sterile inflammatory response triggered by tissue damage is mediated through a multi-protein complex called the inflammasome [7, 21]. In MIRI, the NLRP3 inflammasome is upregulated in CFs and may be a prominent factor in the development of the infarct area in I/R [8]. NLRP3, an important member of the NOD-like receptor family, can form the NLRP3 inflammasome together with ASC and Caspase-1. NLRP3 can stimulate the activation of caspase-1 and promote the release of IL-1β and IL-18 [7]. In our present study, the NLRP3 inflammasome, as expected, was increased in H/R injury (Fig. 5). The activation of Caspase-1 may induce the release of IL-1β and IL-18 by hydrolyzing pro-IL-1β and pro-IL-18 (Fig. 5a, b). The results were further validated at the genetic level (Fig. 5c). Inflammatory cytokines play an important role in the inflammatory response to MIRI, and IL-1β is the key cytokine in the inflammatory response. Monocytes/macrophages and neutrophils migrate to the ischemic myocardium, amplifying the inflammatory reaction within the ischemic heart and inflicting further damage [22–26]. Inhibition of the NLRP3 inflammasome limits the inflammatory injury following MIRI in mice [27].

DEX is a specific agonist of α2-adrenoceptor that has multiple pharmacological functions, such as sedation, analgesia, anxiolysis and maintenance of hemodynamic stability [28]. In addition, DEX has been shown to inhibit the release of proinflammatory cytokines [29, 30], attenuate apoptosis [31, 32], and reduce reactive oxygen species (ROS) production [17, 33]. However, signals inducing the formation and activation of the inflammasome involve the generation of oxidative stress [34]. Excessive ROS production may be crucially involved in the activation of NLRP3 inflammasomes, leading to I/R-induced inflammatory responses [35, 36]. Therefore, we hypothesized that DEX alleviates MIRI by reducing the formation of the NLRP3 inflammasome. As expected, DEX pretreatment significantly suppressed the activation of the NLRP3 inflammasome in CFs (Fig. 6). Thus, the production of inflammatory factors, such as IL-1β, IL-18, and TNF-α, was reduced (Fig. 5c). TNF-α has emerged as significant contributors to myocardial dysfunction [37]. Compelling evidence have identified the nuclear factor-κB (NF-κB) as a key regulator of TNF-α gene activation [38]. And signals provided by NF-κB activators are necessary during the activation of NLRP3 inflammasome [39]. And signals provided by NF-κB activators are necessary during the activation of NLRP3 inflammasome [39]. Studies have shown that Dex inhibits the NF-κB pathway and NLRP3 inflammasome to attenuate papain-induced osteoarthritis in rats [40]. Our research shows that Dex can reduce the production of TNF-α induced by H/R injury in CFs. But whether it is related to NF-κB remains to be verified. Consistent results were obtained at the genetic level. Studies have shown that TNF is an important transcriptional regulator of NLRP3 inflammasome components in murine inflammasomopathies [41]. Therefore, we observed the effect of Dex on NLRP3 mRNA, and studies have shown that Dex inhibits the production of NLRP3 mRNA induced by H/R injury. But the specific mechanism needs to be verified. To further investigate whether DEX inhibits inflammasome activation through NLRP3, thereby attenuating I/R injury, we performed in vitro experiments in CFs treated with MCC950. MCC950 is a potent selective NLRP3 inhibitor that is active both in mice in vivo and in human cells ex vivo [35]. The study found that the inhibition of the NLRP3 inflammasome by DEX pretreatment was consistent with the effect of MCC950. In addition, we also observed the combined effect of DEX and MCC950 pretreatment on the NLRP3 inflammasome in CFs. The results showed that the combined effect of DEX and MCC950 was consistent with their separate effects, further indicating that NLRP3 is a molecular target for DEX-mediated inhibition of NLRP3 inflammasome activation. Therefore, these results indicate that DEX pretreatment inhibits the activation of the NLRP3 inflammasome by acting on NLRP3.

Cardiac function is determined by the collective and dynamic interaction of various cell types and the extracellular matrix that composes the heart [42]. Clearly, CFs play an important role in signaling through their ability to respond to a wide variety of chemical signals that are involved in the paracrine and autocrine regulation of cardiac function [42]. Studies have shown that CFs protect CMs from fatal injury in heart ischemia-reperfusion injury [43]. To further investigate the effects of DEX-pretreated CFs on CMs, we performed a coculture experiment. We found that DEX reduced cell apoptosis by suppressing H/R-induced NLRP3 inflammasome activation in CFs. Research has shown that NLRP3 does not play a role in acute cardiac infarction due to low cardiac expression [44]. This is consistent with our findings; the NLRP3 inflammasome is mainly expressed in CFs not CMs. Therefore, the NLRP3 inhibitor MCC950 did not affect apoptosis in CMs (Fig. 7). Research has shown that DEX attenuates H/R injury in primary neonatal rat CMs [18]. We hypothesize that DEX-mediated protection in CMs is independent of the NLRP3 inflammasome. The role of NLRP3 in MIRI may therefore be more complex than originally thought: on the one hand, NLRP3 inflammasome activation during myocardial ischemia reperfusion is cardioprotective [45], while on the other hand, it may promote further CM loss by inducing pyroptosis, thus leading to greater infarct size and promoting adverse remodeling by inducing the release of IL-1β and IL-18 [46]. This hypothesis needs to be tested in further experiments.

There are some limitations of the present study. Although it has been demonstrated in cells that DEX reduces MIRI by inhibiting the formation of NLRP3 inflammasomes, no functional and in vivo experiments have been conducted, and the upstream signaling pathway is not yet clear, which needs to be investigated further.

## Conclusion

DEX can suppress H/R-induced NLRP3 inflammasome activation in CFs, alleviate MIRI and attenuate the inflammatory response. Future studies evaluating the cardioprotective effects of DEX in the clinical setting of MIRI are warranted.

## Supplementary Information

**Additional file 1.**

## Data Availability

The datasets used and/or analyzed in the current study are available from the corresponding author upon reasonable request.

## Abbreviations

DEX: Dexmedetomidine
AMI: Acute myocardial infarction
MIRI: Myocardial ischemia-reperfusion injury
H/R: Hypoxia/reoxygenation
NLRP3: NOD-, LRR- and pyrin domain-containing protein 3
ASC: Apoptosis-associated speck-like protein containing a CARD
CM: Cardiomyocyte
CF: Cardiac fibroblast
