# Correction to: Dexmedetomidine attenuates myocardial ischemia-reperfusion injury in vitro by inhibiting NLRP3 Inflammasome activation

**DOI:** 10.1186/s12871-021-01356-z

**Published:** 2021-05-10

**Authors:** Yaru Huang, Xiaotong Sun, Zhaodong Juan, Rui Zhang, Ruoguo Wang, Shuqi Meng, Jiajia Zhou, Yan Li, Keyou Xu, Keliang Xie

**Affiliations:** 1 Shandong Provincial Medicine and Health Key Laboratory of Clinical Anesthesia, School of Anesthesiology, Weifang Medical University, No. 7166, Baotong West Street, Weicheng District, Weifang, 261021 China; 2 Department of Pain, Affiliated Hospital of Weifang Medical University, Weifang, 261000 China

**Correction to: BMC Anesthesiol 21, 104 (2021)**

**https://doi.org/10.1186/s12871-021-01334-5**

In the article “Dexmedetomidine attenuates myocardial ischemia-reperfusion injury in vitro by inhibiting NLRP3 Inflammasome activation. *BMC ANESTHESIOL* 2021, 21(1):104” [1] 1uM MCC950 is corrected to 1 μM MCC950 in the Fig. 1a on page 3 of 12.

**Fig. 1:**
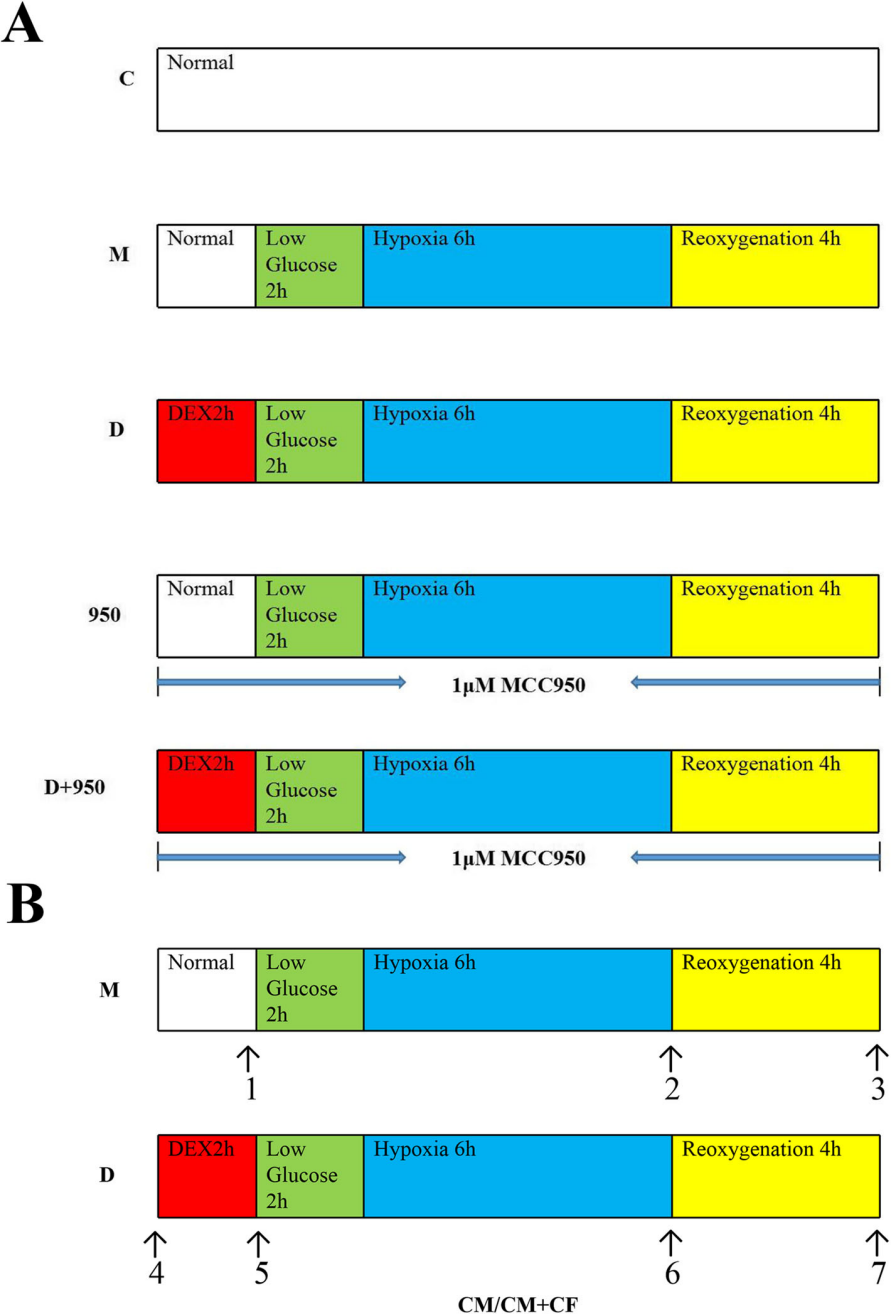
Experimental protocol and four time points for detecting the beating rate of cardiomyocytes (CMs). **a** Experimental protocols. Cardiac fibroblasts (CFs), cardiomyocytes (CMs) and cocultured CMs and CFs (CMs + CFs) were randomly assigned to one of five groups: 1) C group, in which the cells were incubated under normal conditions in a CO2 incubator; 2) M group, in which the cells were exposed to hypoxia/ reoxygenation as we described above; 3) D group, in which the cells were pretreated with 1 μg/ml dexmedetomidine (DEX) 2 h before hypoxia/reoxygenation; 4) 950 group, in which the cells were treated with 1 μM MCC950 during hypoxia/reoxygenation and 2 h before it; and 5) D + 950 group, in which the cells were treated with 1 μM MCC950 during DEX preconditioning and hypoxia/reoxygenation,. **b** Four time points for detecting the myocardial cell beat frequency of cocultured CMs and CFs (CMs + CFs). We detected the beat frequency of CMs at these four time points as indicated by the arrow. 1–2 (group M): 1, normal; 2, after hypoxia/reoxygenation. 3–4 (group D): 3, normal; 4, after hypoxia/reoxygenation. DEX, dexmedetomidine; CMs, cardiomyocytes; CFs, cardiac fibroblasts; CMs + CFs, cocultured cardiomyocytes and cardiac fibroblasts; MCC950, a potent selective NLRP3 inhibitor

## References

[1] Huang Y, Sun X, Juan Z, Zhang R, Wang R, Meng S, Zhou J, Li Y, Xu K, Xie K (2021). Dexmedetomidine attenuates myocardial ischemia-reperfusion injury in vitro by inhibiting NLRP3 Inflammasome activation. BMC Anesthesiol.

