# Supplementary material for: Dexmedetomidine attenuates myocardial ischemia-reperfusion injury in vitro by inhibiting NLRP3 Inflammasome activation
Source: BMC Anesthesiol. 2021 Apr 6;21:104. doi: 10.1186/s12871-021-01334-5 (PMC8022424; doi:10.1186/s12871-021-01334-5)
Supplement: Supplementary file 1 — Additional file 1. [file 12871_2021_1334_MOESM1_ESM.doc]

**Dexmedetomidine Attenuates Myocardial Ischemia-Reperfusion Injury in Vitro by Inhibiting NLRP3 Inflammasome Activation**

Yaru Huang^a,1^, Xiaotong Sun^a,1^, Zhaodong Juan^a,^*, Rui Zhang^a^, Ruoguo Wang^b^, Shuqi Meng^a^, Jiajia Zhou^a^, Yan Li^a^ , Keyou Xu^a^, Keliang Xie^a,^*

*^a^Shandong Provincial Medicine and Health Key Laboratory of Clinical Anesthesia,* *College of Anesthesiology, Weifang Medical University, Weifang 261021, China*

*^b^Department of Pain, Affiliated Hospital of Weifang Medical University, Weifang 261000, China*

^1^These authors contributed equally to this manuscript.

*Corresponding authors: College of Anesthesiology, Weifang Medical University, No. 7166, Baotong West Street, Weicheng District, Weifang 261021, China.

E-mail: [juanzd@wfmc.edu.cn](mailto:juanzd@wfmc.edu.cn) or [xiekeliang2009@hotmail.com](mailto:xiekeliang2009@hotmail.com).





**Supplementary Figure 1** The identified wells represent NLRP3 and other wells are related to another studies. The blots are cropped according to the position marked by the red rectangle.





**Supplementary Figure 2** The identified wells represent ASC and other wells are related to another studies. The blots are cropped according to the position marked by the red rectangle.







**Supplementary Figure 3** The identified wells represent GAPDH and other wells are related to another studies. The blots are cropped according to the position marked by the red rectangle. The second picture is the first picture exposed together with the developing board, which proves that the first picture is the original picture.







**Supplementary Figure 4** The identified wells represent Cleaved-caspase1 and other wells are related to another studies. The blots are cropped according to the position marked by the red rectangle. The second picture is the first picture exposed together with the developing board, which proves that the first picture is the original picture.





**Supplementary Figure 5** The identified wells represent Caspase-1 and other wells are related to another studies. The blots are cropped according to the position marked by the red rectangle.







**Supplementary Figure 6** The identified wells represent IL-1β and other wells are related to another studies. The blots are cropped according to the position marked by the red rectangle. The second picture is the first picture exposed together with the developing board, which proves that the first picture is the original picture.





**Supplementary Figure 7** The identified wells represent Bcl2 in cardiac fibroblasts (CFs). The blots are cropped according to the position marked by the red rectangle.







**Supplementary Figure 8** The identified wells represent BAX in cardiac fibroblasts (CFs) and other wells are related to another studies. The blots are cropped according to the position marked by the red rectangle. The second picture is the first picture exposed together with the developing board, which proves that the first picture is the original picture.







**Supplementary Figure 9** The identified wells represent GAPDH in cardiac fibroblasts (CFs). The blots are cropped according to the position marked by the red rectangle. The second picture is the first picture exposed together with the developing board, which proves that the first picture is the original picture.





**Supplementary Figure 10** he identified wells represent Bcl2 in cardiomyocytes (CMs). The blots are cropped according to the position marked by the red rectangle.





**Supplementary Figure 11** he identified wells represent BAX in cardiomyocytes (CMs). The blots are cropped according to the position marked by the red rectangle.





**Supplementary Figure 12** The identified wells represent GAPDH in cardiomyocytes (CMs). The blots are cropped according to the position marked by the red rectangle.







**Supplementary Figure 13** The identified wells represent Bcl2 in the co-cultured cells of CMs and CFs (CM+CFs). The blots are cropped according to the position marked by the red rectangle. The second picture is the first picture exposed together with the developing board, which proves that the first picture is the original picture.







**Supplementary Figure 14** The identified wells represent BAX in the co-cultured cells of CMs and CFs (CM+CFs). The blots are cropped according to the position marked by the red rectangle. The second picture is the first picture exposed together with the developing board, which proves that the first picture is the original picture.





**Supplementary Figure 15** The identified wells represent GAPDH in the co-cultured cells of CMs and CFs (CM+CFs). The blots are cropped according to the position marked by the red rectangle.
